# Association between oral mucosal lesions and xerostomia: a cross-sectional study in a Brazilian population sample

**DOI:** 10.1590/1807-3107bor-2025.vol39.036

**Published:** 2025-04-04

**Authors:** Soraya de Mattos Camargo GROSSMANN, Luís Cláudio Santos PRADO, Lorena de Andrade e SOUZA, Dayane Priscila DOMINGUES, Fábio Fernandes Borém BRUZINGA, Laura Cascão LOPES, Giovanna Ribeiro SOUTO

**Affiliations:** (a) Pontifícia Universidade Católica de Minas Gerais – PUC-Minas, Department of Dentistry, Belo Horizonte, MG, Brazil.; (b) Universidade Vale do Rio Verde – UninCor, Três Corações, MG, Brazil.

**Keywords:** Epidemiology, Mouth Diseases, Mouth Mucosa, Diagnosis, Oral, Xerostomia

## Abstract

This study aimed to establish the frequency of oral mucosal conditions and xerostomia, identify a possible association between them, and verify their associated factors from a sample of the population of Três Corações, Brazil. A cross-sectional study was conducted with volunteers without age restriction. To evaluate the presence of oral mucosal conditions, an intra-oral examination was performed and a clinical diagnosis was established based on the official classifications of oral diseases. The report of xerostomia was identified by a validated questionnaire completed during anamnesis. Descriptive and association statistics were performed using a significance level of 5%. A total of 1,052 volunteers were evaluated. Oral mucosal lesions were observed in 42.11%, variations of normal structures in 38.50%, and xerostomia in 60.64%. Women were more affected than men, particularly women aged 20-49. Xerostomia was not found to be associated to oral mucosal conditions in general (p > 0.05); however, inflammatory fibrous hyperplasia (27.99%) and oral candidiasis (24.38%), the most prevalent lesions in the study, were associated with xerostomia (p < 0.001 and p = 0.006, respectively) and denture use (p = 0.025 and p < 0.001, respectively). Use of tobacco and alcohol intake were not associated with the presence of oral lesions (p = 0.319 and p = 0.739, respectively). The findings of this study are important for determining the prevalence of oral conditions and xerostomia in the general population, serving as a baseline for further investigations into the association between xerostomia, inflammatory fibrous hyperplasia, and oral candidiasis.

## Introduction

Several epidemiological studies have investigated the prevalence of oral mucosal lesions in the world, with discrepant results. Epidemiological surveys are very important for determining public health plans to prevent disease, but epidemiological studies investigating xerostomia and oral mucosal condition (OMC), including oral mucosal lesions (OML) and variations of normal structures, are scarce.

The worldwide prevalence of OMC ranges from 9.4% to 83.6%.^1-7^ In Brazil, the prevalence of OMC ranges from 27.7% to 86.4%.^8-12^ Women and people in their 40s are the most affected,^13,14^ although other age ranges have been found to be more affected in other Brazilian studies.^15^ Different types of OMC have been observed in the literature, with the more frequent ones being inflammatory fibrous hyperplasia (30.6%), denture stomatitis (23.0%), fibroma (21.3%), hyperkeratosis (19.6%), melanin spots (16.8%), candidiasis (14.3%), mucoceles (9.5%), Fordyce granules (8.8%), coated tongue (8.8%), hemangioma (8.4%), ulcers (8.4%), and nonspecific chronic inflammation (6.3%).^9,12-15^

Studies investigating xerostomia in the general population have been even more scarce. A prevalence of 24.8% of xerostomia was observed in a population over 18 years of age, and xerostomia was associated with use of medications (35.9%) and systemic diseases (i.e. systemic arterial hypertension – 27.6%, and diabetes mellitus – 17.3%) in patients over 50.^16^ Saliva plays an important role in maintaining oral health by providing oral lubrication, protecting against microorganisms, and repairing the oral mucosa. Moreover, saliva presents the buffering capacity necessary for dental remineralization.^17-20^

Considering the importance of epidemiological studies in identifying health determinants and guiding public policies focused on oral health, and seeking to identify the possible association between OMC and xerostomia, the aim of this study was to evaluate the prevalence of xerostomia and OMC in a Brazilian population sample and identify their associated factors.

## Methods

### Study design and sample

Following the approval by the Ethics Committee on Research of Universidade Vale do Rio Verde (protocol No. 229.299), a cross-sectional study was conducted over an 18-month period in the city of Três Corações, an important dentistry reference center in the state of Minas Gerais, Brazil. The study used a convenience sample of adults and children who participated in oral cancer prevention campaigns and were treated in both public and private dental care centers, including factory workers, schoolchildren, and military personnel. The eligibility criteria for inclusion in the study were voluntary participation, residence in Três Corações, age between 0 and 90 years, and the signing of a free and informed consent form. For participants under the age of 18, the consent of their legal guardians was obtained. The exclusion criterion was not residing in this city.

The sample was calculated considering the prevalence of OMC described by Henrique et al.^11^ and based on population data from the Brazilian Institute of Geography and Statistics (IBGE) (SE of 3% and confidence interval level of 95%). Thus, the sample size was 1028 subjects, considering the proportion estimation formula.

### Data collection

The study used questionnaires and intraoral examination to collect data regarding sample characterization, presence of OMC, and xerostomia. Data collection was performed by undergraduate students and professors of the dentistry course of the Universidade Vale do Rio Verde, who were previously trained to perform the intraoral examination and apply the questionnaires. The Kappa test, performed in duplicate, showed almost perfect inter-rater agreement.

Demographical data, tobacco usage, and alcohol intake were obtained from the anamnesis. The history of tobacco was obtained based on criteria defined by Shulman et al.^2^ The presence of xerostomia was recorded through a previously validated questionnaire and, when present, it was classified as mild, moderate, or severe as previously described by Ferreiro et al.^21^ For participants with xerostomia, artificial saliva gel was prescribed to prevent symptoms.

To assess the presence of OMC, only the clinical diagnosis was made, without histopathological confirmation. For this, an intraoral examination was performed in a private area under artificial lighting, with a wooden spatula, following the protocol for universal biological safety procedures. To establish the clinical diagnosis of OMC, the criteria of the World Health Organization^22^proposed by Axéll^23^was applied. The oral examinations were performed following the sequence described by the World Health Organization.^24^ As dental caries or endodontic and inflammatory periodontal diseases cannot be evaluated objectively, these alterations were excluded from the study. The presence of OML and/or variations of normal structures were considered OMC. When more than one OMC was observed, all conditions were recorded. For cases that required biopsy and histopathologic exam for final diagnosis, the patients were forwarded to the Department of Oral Pathology of Universidade Vale do Rio Verde.

### Statistical analysis

The data were summarized through absolute and relative frequencies. The Fisher exact test was used to evaluate the association between xerostomia, OMC, and associated factors (i.e. alcohol intake, tobacco use, and wearing a denture). The same test was used to evaluate the association between the most frequent OML, the presence of xerostomia, and the wearing of denture. Statistical analysis was performed with Epi Info™ (version 7.2; Centers for Disease Control and Prevention, Atlanta, USA). The level of significance was set at 0.05.

## Results

A total of 1,052 participants were examined: 617 (58.65%) women and 435 (41.35%) men. The median age was 38.4 years (range from 0 to 90 years), with the third decade of life being the most frequent with 229 (21.77%) individuals.

OMCs were identified in 848 (80.61%) volunteers, with 443 (42.11%) presenting OML and 405 (38.50%) presenting variations of normal structures. Of all participants, 126 (11.98%) presented both OML and variations of normal structures. OMCs were more prevalent in women aged 31 to 50 years. Table 1 summarizes the findings of OML, variations of normal structures, and xerostomia according to gender and age group.

**Table 1:** Prevalence of oral mucosal lesions, variations of normal structures, and xerostomia according to gender and age group.

Variables	Gender		Age group (years)									Total
	Male	Female	0–10	11–20	21–30	31–40	41–50	51–60	61– 70	71–80	81–90	n (%)
	n (%)	n (%)	n (%)	n (%)	n (%)	n (%)	n (%)	n (%)	n (%)	n (%)	n (%)	
Oral mucosa lesions												
Present	201 (19.11)	242 (23.00)	27 (2.57)	42 (3.99)	64 (6.08)	76 (7.22)	87 (8.27)	60 (5.70)	57 (5.42)	15 (1.43)	15 (1.43)	443 (42.11)
Absent	234 (22.24)	375 (35.65)	54 (5.13)	57 (5.42)	165 (15.69)	105 (9.98)	93 (8.84)	66 (6.28)	39 (3.70)	24 (2.28)	6 (0.57)	609 (57.89)
Variation of normal structures												
Present	165 (15.68)	240 (22.82)	39 (3.71)	30 (2.85)	69 (6.56)	78 (7.41)	66 (6.28)	51 (4.85)	39 (3.70)	24 (2.29)	9 (0.85)	405 (38.50)
Absent	270 (25.67)	377 (35.83)	42 (3.99)	69 (6.56)	160 (15.21)	103 (9.79)	114 (10.83)	75 (7.13)	57 (5.42)	15 (1.42)	12 (1.15)	647 (61.50)
Xerostomia												
Present	242 (23.00)	396 (37.64)	48 (4.56)	51 (4.85)	154 (14.64)	100 (9.50)	117 (11.12)	69 (6.56)	60 (5.70)	21 (2.00)	18 (1.71)	638 (60.64)
Absent	168 (15.97)	207 (19.68)	27 (2.57)	45 (4.27)	72 (6.84)	75 (7.13)	51 (4.85)	51 (4.86)	33 (3.13)	18 (1.71)	3 (0.29)	375 (35.65)
Not informed	25 (2.38)	14 (1.33)	6 (0.57)	3 (0.29)	3 (0.29)	6 (0.47)	12 (1.14)	6 (0.56)	3 (0.29)	0 (0.00)	0 (0.00)	39 (3.71)
Total	435 (41.35)	617 (58.65)	81 (7.70)	99 (9.41)	229 (21.77)	181 (17.20)	180 (17.11)	126 (11.98)	96 (9.12)	39 (3.71)	21 (2.00)	1.052 (100.00)

n: absolute frequency; % relative frequency.

Xerostomia was observed in 638 (60.64%) individuals, with especially high representation in women (396 cases – 37.64%) (Table 1). Xerostomia was classified as mild in 447 cases (70.06%), moderate in 170 cases (26.65%), and severe in 21 cases (3.29%).

The wearing of dentures was reported in 222 participants (21.10%) while 117 (27.47%) reported alcohol intake and 90 (8.60%) reported smoking history. Presence of OMC was not associated with either alcohol consumption or smoking history (p > 0.05), but it was associated with the use of denture (p < 0.001) (Table 2).

**Table 2:** Relationship between presence of oral mucosal lesions and alcohol intake, use of tobacco, and use of denture.

Oral mucosal lesions	Alcohol intake		p-value*	Use of tobacco		p-value*	Use of denture		p-value*
	Yes	No		Yes	No		Yes	No	
	n (%)	n (%)		n (%)	n (%)		n (%)	n (%)	
Present	42 (9.86)	129 (30.29)	0.319	36 (3.44)	401 (38.34)	0.739	129 (12.26)	314 (29.85)	**< 0.001**
Absent	75 (17.61)	180 (42.25)		54 (5.16)	555 (53.06)		93 (8.84)	516 (49.05)	
Total	117 (27.47)	309 (72.54)		90 (8.60)	956 (91.40)		222 (21.10)	830 (78.90)	

n: absolute frequency; % relative frequency; *Fisher exact test; Valid value: Alcohol intake (426) – Use of tobacco (1,046) – Use of denture (1,052)

Fourteen different OMLs were observed in the sample. The most common types included inflammatory fibrous hyperplasia (124 – 27.99%), oral candidiasis (108 – 24.38%), herpes labialis (39 – 8.80%), and leukoplakia (30 – 6.77%) (Table 3). The other OMLs observed are shown in Table 3, summarized by gender and age group. Regarding variations of normal structures, eight different types could be observed in 396 participants (37.64%), with no differences related to gender. The most prevalent conditions were lingual varicosities (141 – 35.61%), Fordyce’s spots (92 – 23.23%), and fissured tongue (57 – 14.39%). No association was found between presence of xerostomia and OML or variations of normal structures (p > 0.05) (Table 4).

**Table 3:** Prevalence of oral mucosal lesions by gender and age group.

Oral mucosal lesions	Gender		Age group (years)									Total
	Male	Female	0–10	11–20	21–30	31–40	41–50	51–60	61– 70	71–80	81–90	
	n (%)											
Inflammatory fibrous hyperplasia	54 (12.19)	70 (15.80)	6 (1.35)	12 (2.71)	28 (6.32)	6 (1.35)	24 (5.42)	15 (3.39)	24 (5.42)	3 (0.68)	6 (1.35)	124 (27.99)
Oral candidiasis	45 (10.16)	63 (14.22)	6 (1.35)	6 (1.35)	12 (2.71)	24 (5.42)	24 (5.42)	15 (3.39)	12 (2.71)	6 (1.35)	3 (0.68)	108 (24.38)
Herpes labialis	21 (4.74)	18 (4.06)	0 (0.00)	9 (2.03)	3 (0.68)	9 (2.03)	9 (2.03)	6 (1.35)	3 (0.68)	0 (0.00)	0 (0.00)	39 (8.80)
Leukoplakia	12 (2.71)	18 (4.06)	3 (0.68)	0 (0.00)	0 (0.00)	15 (3.38)	6 (1.35)	0 (0.00)	3 (0.68)	3 (0.68)	0 (0.00)	30 (6.77)
Petechia	12 (2.71)	15 (3.38)	3 (0.67)	6 (1.36)	6 (1.36)	0 (0.00)	6 (1.35)	6 (1.35)	0 (0.00)	0 (0.00)	0 (0.00)	27 (6.09)
Traumatic ulceration	12 (2.71)	12 (2.71)	3 (0.68)	3 (0.68)	3 (0.68)	6 (1.35)	6 (1.35)	3 (0.68)	0 (0.00)	0 (0.00)	0 (0.00)	24 (5.42)
Actinic cheilosis	15 (3.38)	6 (1.36)	0 (0.00)	3 (0.67)	3 (0.67)	3 (0.68)	6 (1.36)	0 (0.00)	3 (0.68)	0 (0.00)	3 (0.68)	21 (4.74)
Pigmented injuries	9 (2.03)	7 (1.59)	3 (0.68)	0 (0.00)	0 (0.00)	4 (0.90)	0 (0.00)	3 (0.68)	3 (0.68)	0 (0.00)	3 (0.68)	16 (3.62)
Pyogenic granuloma	0 (0.00)	12 (2.71)	0 (0.00)	0 (0.00)	3 (0.68)	6 (1.35)	3 (0.68)	0 (0.00)	0 (0.00)	0 (0.00)	0 (0.00)	12 (2.71)
Exostosis	6 (1.35)	3 (0.68)	0 (0.00)	0 (0.00)	0 (0.00)	3 (0.67)	0 (0.00)	3 (0.68)	3 (0.68)	0 (0.00)	0 (0.00)	9 (2.03)
Giant cell fibroma	3 (0.68)	6 (1.35)	3 (0.68)	0 (0.00)	0 (0.00)	0 (0.00)	3 (0.67)	0 (0.00)	3 (0.68)	0 (0.00)	0 (0.00)	9 (2.03)
Mucocele	6 (1.35)	3 (0.68)	0 (0.00)	0 (0.00)	3 (0.68)	0 (0.00)	0 (0.00)	3 (0.67)	3 (0.68)	0 (0.00)	0 (0.00)	9 (2.03)
Sialadenitis	3 (0.68)	6 (1.35)	0 (0.00)	3 (0.68)	0 (0.00)	0 (0.00)	0 (0.00)	3 (0.67)	0 (0.00)	3 (0.68)	0 (0.00)	9 (2.03)
Frictional keratosis	3 (0.68)	3 (0.68)	0 (0.00)	0 (0.00)	3 (0.68)	0 (0.00)	0 (0.00)	3 (0.68)	0 (0.00)	0 (0.00)	0 (0.00)	6 (1.36)
Total	201 (45.37)	242 (54.63)	27 (6.09)	42 (9.48)	64 (14.46)	76 (17.13)	87 (19.63)	60 (13.54)	57 (12.89)	15 (3.39)	15 (3.39)	443 (100.00)

n: absolute frequency; % relative frequency.

**Table 4:** Association between xerostomia and variations of normal structures and oral mucosal lesions.

Xerostomia	Variants of normal structures		p-value*	Oral mucosal lesion		p-value*
	Present	Absent		Present	Absent	
	n (%)	n (%)		n (%)	n (%)	
Present	261 (25.77)	377 (37.21)	0.125	266 (26.26)	372 (36.72)	0.791
Absent	135 (13.33)	240 (23.69)		153 (15.10)	222 (21.92)	
Total	396 (39.10)	617 (60.90)		419 (41.36)	594 (58.64)	

n: absolute frequency; % relative frequency; *Fisher exact test; Valid value (1,013).

The two most frequent OMLs (inflammatory fibrous hyperplasia and oral candidiasis) were associated with xerostomia and use of dentures (Table 5). The data show that inflammatory fibrous hyperplasia and oral candidiasis were more frequent in individuals who used dentures (p = 0.025 and p < 0.001, respectively). Similarly, individuals with inflammatory fibrous hyperplasia or oral candidiasis presented more reports of xerostomia (p < 0.001 and p = 0.006, respectively).

**Table 5:** Association between inflammatory fibrous hyperplasia and oral candidiasis with use of denture and xerostomia.

Variables	Use of denture		p-value*	Xerostomia		p-value*
	Yes	No		Present	Absent	
	n (%)	n (%)		n (%)	n (%)	
Inflammatory fibrous hyperplasia						
Present	36 (3.42)	88 (8.36)	0.025	91 (8.98)	24 (2.37)	< 0.001
Absent	186 (17.68)	742 (70.54)		547 (54.00)	351 (34.65)	
Oral candidiasis						
Present	48 (4.56)	60 (5.71)	< 0.001	81 (8.00)	27 (2.67)	0.006
Absent	174 (16.54)	770 (73.19)		557 (54.98)	348 (34.35)	

n: absolute frequency; % relative frequency; *Fisher exact test; Valid values for use of denture (1052) and xerostomia (1013).

## Discussion

There are few population-based studies that have investigated the prevalence of OML in adults.^2,23^ Comparing the results from different epidemiological studies about OMC in different populations is not easy, mainly due variations in the methodologies and the geographical characteristics of the samples. The prevalence of OMC in our sample was higher than 80%, which is close to the findings of Vieira et al.,^9^ who found a prevalence of 75.3% in a population of similar geographical and socio-economic characteristics. However, results from population studies differ from those conducted within educational institutions and health reference centers.^1-5^ Another important factor is the analysis of variations of normal structures as part of the sum of OML.^11^ In the present study, these conditions were evaluated separately from OML.

In the reviewed literature, women are more affected by OML than men, which aligns with the results observed in this study.^11,12^ However, one study reported that OML was more frequent in men.^3^ Different studies have shown that the prevalence of xerostomia in the general population is about 20%,^25,26^ different from the results of the present study, in which 60.64% of the sample had xerostomia. Differences in xerostomia evaluation methodology may justify such disagreement. A higher prevalence of xerostomia has been observed in patients aged 50 years or older,^16^ while just over 70% of people with xerostomia were younger than 50 years in our sample. Those in their 20s were also more affected (24.14% of affected individuals).

In this study, were observed in 405 participants (38.50%). A total of 261 participants (25.77%) had both variations of normal structures and xerostomia; however, no statistical association was observed between the two. Similarly, variations of normal structures were reported in 35.93% of another Brazilian population^28^. Nevertheless, a separate population study reported a lower prevalence of these variations (e.g., 8.8% prevalence of Fordyce spots and 6.7% of fissured tongue), with some types not observed in the sample (such as lingual varicosities).^9^ This discrepancy may be due to the younger age range of patients in our study, where patients presenting these variations were in the 31–40 age group (7.41%). However, such conditions are more commonly seen in older adults, as emphasized in other studies.^1,6,27^

Inflammatory fibrous hyperplasia was the most prevalent OML, followed by oral candidiasis associated to the use of denture. This finding agrees with the literature, which classifies the use of denture as an etiological factor for the appearance of these types of lesions.^2,6,29^ The present study is a cross-sectional study, so a causal relationship cannot be established between the variables evaluated. Moreover, the lesions were diagnosed only by clinical and epidemiological criteria, which may influence the findings, being a limitation of the study.

The general analysis showed no association between xerostomia and OML, but isolated analyses of the two most frequent lesions showed that both were associated with dry mouth. Although dry mouth has several causes, hyposalivation is the most common reason. It is known that salivary flow reduction predisposes the development of oral candidiasis^30^. Furthermore, the reduction of salivary flow in patients with a report of xerostomia has been shown to be related to the formation and increase in the number of *Candida sp* colonies.^31^

In general, xerostomia was not associated to the presence of OML, and only 26.26% of participants presented both conditions. In contrast, another study found that the reduction of salivary flow and xerostomia were related to the presence of OML. However, that study only included elderly individuals, with different systemic conditions and their drug treatment, which can lead to confounding factors.^27^ In the present study, individuals aged 0–90 years were evaluated, which makes it possible to control for age-related confounding factors.

Fourteen different OML clinical diagnoses were observed in this study, including proliferative lesions, fungal and viral infections, and traumatic processes. When factors associated with OML were evaluated, alcohol intake and use of tobacco did not influence the appearance of lesions, unlike the study of Lynge Pedersen et al.,^27^ who observed a two-fold increase risk of oral injuries due to smoking. In the same study, alcohol use was also not associated with the presence of OML.

## Conclusion

Considering the high overall prevalence of xerostomia in our sample, further epidemiological studies in different geographical areas are warranted. As cross-sectional studies cannot definitively establish causal association between factors and diseases, the association between OMC and xerostomia is still a debatable issue. Thus, we suggest that in the presence of xerostomia, possible oral alterations should be investigated, especially in young patients. Our results can be used as baseline data for future studies, considering the scarcity of research investigating the prevalence of OMC in patients with xerostomia.
